# Dexamethasone versus triamcinolone acetonide for the management of postoperative inflammation following cataract surgery: A systematic review and meta-analysis

**DOI:** 10.1097/MD.0000000000050840

**Published:** 2026-10-09

**Authors:** Humayou Ahmed, Muhammad Saad Khan, Zauha Fawad Memon, Serish Bano, Samreen Shahid, Zainab Arif, Syed Ali Hassan, Umarah Lutuf, Wajeeha, Shifa, Aqsa, Muhammad Mohsin Khan, Daniyal Zulfiqar, Maheen Hamid, Manal Al-Dhaheri

**Affiliations:** a Internal Medicine, Dow University of Health Sciences, Karachi, Pakistan; b Internal Medicine, Jinnah Sindh Medical University, Karachi, Pakistan; c Internal Medicine, Peoples University of Medical and Health Sciences for Women, Shaheed Benazirabad, Pakistan; d Internal Medicine, Shaheed Mohtarma Benazir Bhutto Medical College Lyari, Karachi, Pakistan; e Internal Medicine, Karachi Medical and Dental College, Karachi, Pakistan; f Internal Medicine, Liaquat University of Medical and Health Sciences, Jamshoro, Pakistan; g Internal Medicine, Multan Medical and Dental College, Multan, Pakistan; h Internal Medicine, Sana’a University, Sanaa, Yemen.

**Keywords:** cataract surgery, dexamethasone, meta-analysis, postoperative-inflammation, triamcinolone-acetonide

## Abstract

**Background:**

Cataract remains the leading cause of reversible blindness worldwide, particularly in low-resource settings. Effective control of postoperative inflammation is essential to prevent complications such as cystoid macular edema and raised intraocular pressure. Dexamethasone and Triamcinolone acetonide (TCA) are widely used corticosteroids; however, their comparative efficacy remains uncertain. This updated systematic review and meta-analysis aimed to compare their effectiveness following cataract surgery.

**Methods:**

A systematic search of 4 databases (PubMed, Google Scholar, Cochrane Central Library and Scopus) was conducted up to June 2026 following Preferred Reporting Items for Systematic Reviews and Meta-Analyses guidelines (International Prospective Register of Systematic Reviews: CRD420261435380). Randomized controlled trials and prospective studies comparing dexamethasone and TCA in adults undergoing phacoemulsification were included. Risk of bias was assessed using cochrane risk of bias 2 and the Newcastle–Ottawa Scale. Certainty of evidence was evaluated using grading of recommendations assessment, development and evaluation. A random-effects model was used, with heterogeneity assessed by *I*^2^.

**Results:**

Twelve studies (nine randomized controlled trials and 3 non-randomized prospective studies; n = 2003) were included. No significant differences were observed in intraocular pressure (Mean Difference, mm Hg) at any time point up to 3 months. Visual acuity showed modest improvement with TCA at 7 and 28/30 days, though results were inconsistent on sensitivity analysis. No significant differences were found in anterior chamber flare or cells across all time points. A nonsignificant trend toward greater endothelial cell loss was observed with dexamethasone. Overall, evidence certainty was low.

**Conclusion:**

Dexamethasone and TCA demonstrate comparable efficacy and safety in controlling postoperative inflammation after cataract surgery. TCA may offer short-term visual benefits and prolonged action, whereas dexamethasone allows flexible dosing. Clinical decisions should be individualized, considering patient compliance and clinical context.

## 1. Introduction

Cataract is defined as progressive opacification of the crystalline lens which causes a decrease in visual acuity (VA), and is the primary cause of reversible blindness in the world, especially in low and middle-income countries.^[1]^ Although there has been great progress in eye care, cataract is still a significant cause of visual impairment worldwide and impacts millions of people annually. In the world, about 65.2 million people suffer from visual impairment or blindness caused by cataract, most of which can be prevented if treated in time by surgical methods.^[2]^ The treatment is surgical (mainly phacoemulsification) and is one of the most common surgical procedures in the world.^[3,4]^

While the surgeon’s success rate with cataract surgery is extremely high, postoperative inflammation can be an important clinical concern. Surgical manipulation causes a breakdown of the blood–aqueous barrier, which causes inflammation and, if this is not controlled, can result in complications, including cystoid macular edema, increased intraocular pressure (IOP), posterior capsule opacification, posterior synechiae, and secondary glaucoma, all of which will affect visual recovery.^[5,6]^ Hence, the importance of achieving good suppression of postoperative inflammation, to optimize surgical outcomes and maintain visual function.

Among the most commonly used ophthalmic corticosteroids used after cataract surgery is dexamethasone.^[7]^ It has a potent anti-inflammatory activity, which is achieved by blocking several steps of the inflammatory cascade (genomic and non-genomic effect) and thus affecting thousands of inflammatory pathways downstream. The pharmacologic effects make dexamethasone a useful drug in the prevention and treatment of postoperative ocular inflammation.^[8]^

Triamcinolone acetonide (TCA) is an intermediate potency corticosteroid and has a long duration of action, giving a prolonged anti-inflammatory effect after a single dose.^[9]^ In addition to inhibiting inflammatory mediators, TCA decreases vascular permeability, angiogenesis and cellular proliferation, and plays a role in the resolution of macular edema.^[10,11]^ TCA has been used in ophthalmology for over 50 years, and has been successful for its anti-inflammatory effect after phacoemulsification surgery, with some studies reporting that it provides long-term anti-inflammatory action and better postoperative results.^[12,13]^

There are several clinical trials comparing the efficacy of dexamethasone and/or TCA in postoperative inflammation following cataract surgery; however, the relative efficacy of both are still unclear. In general, the anti-inflammatory activity of individual studies has been reported to be similar, and some have indicated that the effects of TCA might be more tolerable or more enduring due to its longer duration of action in the eye.^[14]^ Zhou et al (2024) performed the first systematic review and meta-analysis of the available studies with 7 studies, and found no significant difference between dexamethasone and TCA for IOP, logarithm of the minimum angle of resolution (logMAR) VA, anterior chamber cells, or anterior chamber flare, but found that there were more anterior chamber cells on postoperative day one with TA.^[15]^

This updated Systemic review and meta-analysis aims to assess comparative efficacy of Dexamethasone Versus TCA in the management of postoperative inflammation following cataract surgery with 5 new studies along with 7 studies that were analyzed in previous meta for IOP, VA, Anterior chamber cells, Anterior chamber flare along with an additional outcome of Endothelial cell density (ECD). The rationale of this meta lies on the fact that the 5 newly added studies contribute novel outcomes (including ECD), expand geographic diversity (Egypt, Finland), and extend follow-up to 3 months. Additionally, in contrast to the previous meta-analysis we performed a detailed follow-up analysis of each outcome by postoperative days along with the grading of recommendations assessment, development and evaluation (GRADE) assessment, to better characterize the temporal profile of post-surgery inflammation status and intraocular pressure changes, thereby providing a more comprehensive synthesis of available evidence and illustrating more robust assessment of heterogeneity in order to reevaluate the comparative efficacy of Dexamethasone versus TCA after cataract surgery.

## 2. Material and methods

An updated systematic review and meta-analysis was conducted in accordance with the Preferred Reporting Items for Systematic Reviews and Meta-Analyses guidelines.^[16]^ We did not need ethical approval or patient permission because our research was based on data from published literature. The protocol has been registered with International Prospective Register of Systematic Reviews reg number: *CRD420261435380* and accessible via the following link https://www.crd.york.ac.uk/PROSPERO/view/CRD420261435380

### 2.1. Search strategy and data sources

A comprehensive search was conducted across the following databases without any conditions or restrictions: PubMed (MEDLINE), Google Scholar, Cochrane Central Library and Scopus, using search terms like ((“phacoemulsification” OR “cataract surgery” OR “cataract extraction”) AND (“TCA” OR “triamcinolone” OR “intracameral triamcinolone”) AND (“dexamethasone” OR “topical dexamethasone” OR “dexamethasone eye drop” OR “dexamethasone ophthalmic”) AND (“intraocular pressure” OR “IOP” OR “VA” OR “anterior chamber cells” OR “anterior chamber flare” OR “ECD” OR “endothelial cell count”)) AND (randomized OR randomized OR “randomized controlled trial (RCT)” OR “RCT” OR RCT OR “clinical trial”) to find the relevant articles that have been published till June, 2026. The author also searched for published or unpublished articles on clinicaltrials.gov. A manual search to gather RCTs related to the included research, systematic reviews, and meta-analyses was also conducted later to find any possible relevant studies.

### 2.2. Study selection and eligibility criteria

RCTs and Non-Randomized prospective studies published in English and focused on the effects of Dexamethasone vs TCA following cataract surgery or phacoemulsification patients were eligible for inclusion. Trials including adults above 50 years old were included. Studies needed to report at least one of the following outcomes: IOP, VA, anterior chamber flare, anterior chamber cells, or ECD. Review articles, case reports, and observational studies (such as case-control, retrospective, and cross-sectional studies) were excluded from the review. Studies that were conducted on animals or patients with other conditions or who underwent other surgeries were also excluded.

### 2.3. Data extraction and risk of bias assessment

Two authors used Rayyan AI, an AI-powered online platform for systematic literature review management, to do the screening.^[17]^ Duplicate studies were found and removed from the list. Screening of all the articles was done initially based on the title and abstract, then the remaining articles through the full text. Any disagreements over the inclusion or exclusion of specific studies, the extraction of data, and the interpretation of that data were settled by consensus. If a trial was described in more than 1 publication, only the study with the most comprehensive data was listed. Finally, the same 2 reviewers cross-checked the selected outcomes. A third reviewer arbitrated the final judgement if there were any disputes during the process. Subsequently, the accuracy of these data were cross-checked by the same 2 reviewers. Any differences of opinion were settled by consensus or by having a third reviewer make a decision. For making baseline characteristics and outcome tables, a Microsoft Excel spreadsheet was created, and data from the included RCTs were inserted accordingly. The baseline parameters were as follows: name of first author; country; publication year; number of participants; intervention (Dexamethasone) and comparator (TCA); and data like age, gender, pre-op intraocular pressure, and the location of the operated eye (right/left). The cochrane risk of bias tool 2 was utilized by 1 reviewer to evaluate the quality assessment and risk of bias of each included RCT across 5 domains.^[18]^ For Non-randomized prospective studies, the Newcastle-Ottawa Scale (NOS) was applied to evaluate selection, comparability, and outcome.^[19]^ Before the final analysis, the assessment results were compared, and any differences were settled by consensus.

### 2.4. Primary outcomes

The primary outcomes evaluated in this meta-analysis were:

- IOP changes seen in TCA vs Dexamethasone administration over time intervals of 1 day, 7 days, 28/30 days, and 3 months post-op.

### 2.5. Secondary outcomes

- VA: BCVA = Best-Corrected VA and CDVA = Corrected Distance VA. LogMAR (logarithm of the minimum angle of resolution) is a scientific scale for VA. It was measured over time intervals of 1 day, 7 days and 28/30 days.
- Anterior chamber flare: Defines protein content present in aqueous humor. Higher levels indicate breakdown of the blood-aqueous barrier and leakage of proteins into the aqueous humor. It was measured over time intervals of 1 day, 7 days and 28/30 days.
- Anterior chamber cells: Indicated the number of inflammatory cells (white blood cell) present in the aqueous humor. A marker of inflammation of the anterior chamber. It was measured over time intervals of 1 day, 7 days and 28/30 days.
- ECD: Refers to the number of corneal endothelial cells per square millimeter (cells/mm^2^). The cells do not regenerate. With age, their number decreases.

### 2.6. Statistical analysis

The analysis was performed using Review Manager (v5.4.1). A random-effects model was employed to take into consideration the heterogeneity among the included RCTs. IOP, VA, anterior chamber flare, anterior chamber cells, and ECD, anterior chamber flare, and anterior chamber cells were all continuous results, so mean differences (MD) and standardized mean difference (SMD) with 95% confidence intervals (CIs) were computed to determine them. For visual representation of data, forest plots were created. In cases where substantial heterogeneity was detected (*I*^2^ > 50%), sensitivity analyses were undertaken by sequentially excluding individual studies to identify potential sources contributing to the observed heterogeneity. Low, moderate, and high heterogeneity are indicated by values of 25%, 50%, and 75%, respectively.

### 2.7. Publication bias

We employed funnel plots in Comprehensive Meta-Analysis (version 3.0) to evaluate publication bias.^[20]^

### 2.8. Certainty of evidence

We used a GRADE tool to assess the overall quality and strength of evidence for each primary and secondary outcome.^[21]^

### 2.9. Ethical approval

Ethical approval was not required for this study as this study was done on previously published data.

## 3. Results

### 3.1. Study selection

A total of 86 records were identified from the searched databases (PubMed/MEDLINE, Google Scholar, Cochrane Central Library and Scopus), of which 10 duplicates were removed. 55 records were eliminated for being irrelevant or having an ineligible study design after being screened for titles and abstracts. The eligibility of remaining full-text publications was evaluated. Nine of these studies were disqualified for miscellaneous reasons. Ultimately, the systematic review and meta-analysis contained twelve trials. Of these twelve included studies, 4 are newly identified RCTs and 1 Non-Randomized prospective trial that have not been included in previous meta-analyses on this topic. A detailed flow diagram of the study selection process is provided in Figure 1.

**Figure 1.**
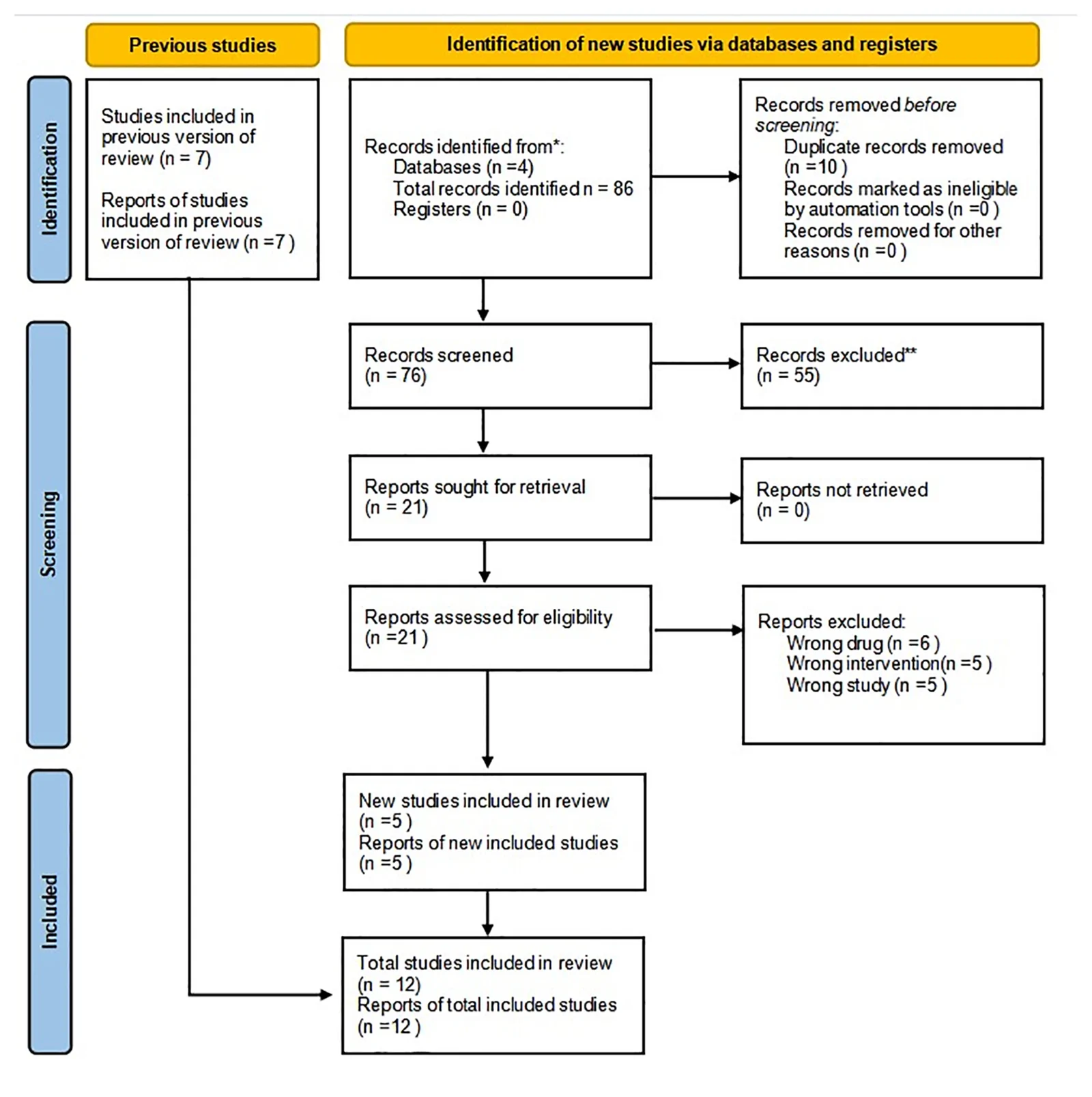
PRISMA flow diagram illustrating the study selection process. PRISMA = Preferred Reporting Items for Systematic Reviews and Meta-Analyses.

### 3.2. Baseline characteristics

Table 1 summarizes the baseline characteristics of participants across the twelve included studies which include 9 RCTsand 3 Non-Randomized prospective studies (Elwakeel 2024, Elkhodary 2023, Choopong 2022, Shaheen 2020, Manzoor 2018, Lindohlm 2018, Rattan 2018, Khan 2016, Gungor 2014, Alam 2013, Wang 2013, and Simaroj 2011) evaluating the effects of Dexamethasone (Dex) versus TCA in patients undergoing phacoemulsification. The table includes data on the first author, publication year, country, number of patients, age, gender distribution, operated eye (right/left), and preoperative IOP.

**Table 1:** Demographic characteristics of the patients.

Study	Yr	Country	No of patients	Age		Gender				Eye				Pre-op IOP (mm Hg)	
				Dex	TCA	Dex		TCA		Right		Left		Dex	TCA
						Male	Female	Male	Female	Dex	TCA	Dex	TCA		
Mahmoud Mostafa H. Elwakeel et al.	2024	Egypt	40 (Dex n = 20, TCA n = 20)	60.700 ± 5.100	60.000 ± 6.096	12	8	7	13	9	12	11	8	NA	NA
M. Elkhodary et al	2023	Egypt	60 (Dex n = 30, TCA n = 30)	56.03 ± 11.44	59.33 ± 9.37	15	15	20	10	16	18	14	12	14.70 ± 3.12	14.47 ± 2.70
Pitipol Choopong et al.	2022	Thailand	140 (Dex n = 70, TCA n = 70)	68.41 ± 7.91	68.21 ± 9.61	23	47	36	34	39	42	31	28	NA	NA
Mumtaz Alam et al.	2013	Pakistan	168 (Dex n = 84, TCA n = 84)	57.23 ± 8.98 years	59.23 ± 10.29	52	32	54	30	NA	NA	NA	NA	14.60 ± 2.36	14.80 ± 2.39
Asif Manzoor et al.						**Male**		**Female**							
	2018	Pakistan	60 (Dex n = 60, TCA n = 30)	56.63 ± 5.87	58.63 ± 7.30	34		26		NA	NA	NA	NA	NA	NA
Sirel Gür Güngör et al.	2014	Turkey	60 (Dex n = 60, TCA n = 30)	71.4 ± 9.4	69.8 ± 10.5	14	16	10	20	NA	NA	NA	NA	15.1 ± 1.75	14.7 ± 1.75
Habibullah Khan et al.	2016	Pakistan	108 (Dex n = 54, TCA n = 54)	58.87 ± 9.69	57.77 ± 8.93	37	17	35	19	NA	NA	NA	NA	14.94 ± 2.24	14.52 ± 2.33
Kamran Haider Shaheen et al.	2020	Pakistan	80 (Dex n = 40, TCA n = 40)	51 ± 4	50 ± 5	29	11	22	18	NA	NA	NA	NA	16.02 ± 2.55	17.14 ± 2.17
Pornchai Simaroj et al.	2011	Thailand	60 (Dex n = 60, TCA n = 30)	65.37 + 7.97	66.77 + 8.29	12	18	12	18	NA	NA	NA	NA	13.81 ± 2.23	13.92 ± 2.25
Bingsong Wang et al. (a)	2013	China	*(Total = 126*) TCA 0.5 mg n = 32, Dex n = 33	65.39 ± 6.08	63.69 ± 9.66	14	19	14	18	NA	NA	NA	NA	27.09 ± 5.89	25.69 ± 6.87
Bingsong Wang et al. (b)			TCA 1 mg n = 31, Dex n = 33	65.39 ± 6.08	62.90 ± 8.74	14	19	16	15	NA	NA	NA	NA	27.09 ± 5.90	28.84 ± 8.05
Bingsong Wang et al. (c)			TCA 2mg n = 30, Dex n = 33	65.39 ± 6.08	64.33 ± 8.27	14	19	17	13	NA	NA	NA	NA	27.09 ± 5.91	26.33 ± 6.09
Juha-Matti Lindholm et al	2019	Finland	101 (Dex n = 50, TCA n = 51)	74.5 ± 6.5	74.7 ± 6.7	16	34	17	34	28	29	22	22	14.0 ± 3.4	15.4 ± 2.9
Susan Amana Rattan et al.	2018	Iraq	1000 (Dex n = 500, TCA n = 500)	59.4 ± 9.04	59.7 ± 8.84	200	300	180	320	NA	NA	NA	NA	14. 49 ± 3. 11	14. 47 ± 3. 10

Dex = Dexamethasone, IOP = intraocular pressure, TCA = triamcinolone Acetonide.

With sample sizes in individual RCTs ranging from 60 to 1000. The total study population across all trials was 2003. Egypt, Thailand, Pakistan, Turkey, China, Finland, and Iraq were the locations of the studies. Participants’ average age was constant across research, usually between 50 and 70 years old, which met the inclusion requirements for individuals over 50. The majority of research indicated a fairly equal gender distribution.

Successful randomization for this crucial parameter was demonstrated by the baseline preoperative IOP being similar between the Dexamethasone and TCA groups in each research, where provided. The laterality of the operated eye (right/left) was also reported as balanced in the studies that provided this data. The baseline characteristics suggest that the compared groups were well-matched at the outset of the included trials, supporting the validity of the subsequent comparative analyses.

### 3.3. Quality assessment

We used the Cochrane cochrane risk of bias 2 to assess the risk of bias in the included RCTs. The summary of the risk of bias assessment is presented in Figure 2. Nine of the twelve included RCTs demonstrated an overall low risk of bias across all 5 domains: bias arising from the randomization process (D1), bias due to deviations from intended interventions (D2), bias due to missing outcome data (D3), bias in measurement of the outcome (D4), and bias in selection of the reported result (D5). Two studies (Wang, 2013 and Simaroj, 2011) were rated as having a high risk of bias. Wang (2013) exhibited concerns specifically in domain D2 (bias in deviations from the intended interventions), while Simaroj (2011) showed issues in domain D2 and D5 (bias in selection of the reported result).

**Figure 2.**
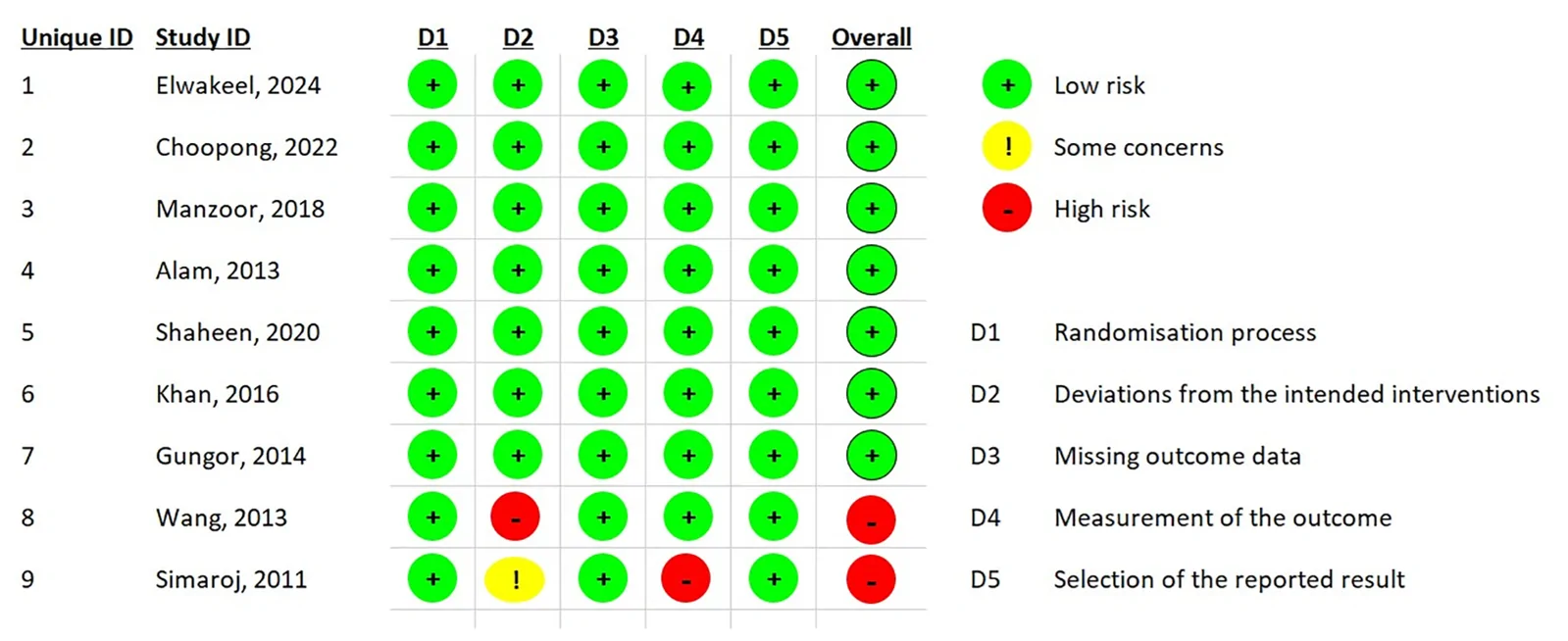
Risk of bias assessment of the 9 included randomized controlled trials using the Cochrane RoB 2 tool.

NOS was used to assess the quality of non-randomized studies included in this review. There are 3 cohort studies included in this review (Elkhodary, 2023; Lindholm, 2018; Rattan, 2018) that were assessed by the NOS. In this review, all three of the included studies received an overall rating of good quality based upon the assessment of three broad categories: the selection of study groups; the comparability of the study groups, including of the relative matching of subjects to each other based upon significant confounding factors; and the ascertainment of the outcomes for the subjects. There was no disagreement between the 2 independent reviewers who assessed the studies, thus demonstrating consistency in the quality assessment of the studies. As for overall star ratings on those three broad categories, all three cohorts received maximum or near maximum stars on both the selection and outcomes categories; and two of them (Lindholm, 2018 and Rattan, 2018) received the highest star rating available for the comparability category. Thus, the majority of the cohorts demonstrated good quality overall due to high quality groups in all three of the broad categories evaluated by the NOS. (Supplementary Table 1, Supplemental Digital Content 6).

### 3.4. Meta-analysis of primary outcomes

#### 3.4.1. Effect of dexamethasone versus TCA on IOP

The pooled analysis of IOP changes at various postoperative time intervals is presented in Figure 3A–D). The results for each prespecified time point are as follows:

**Figure 3.**
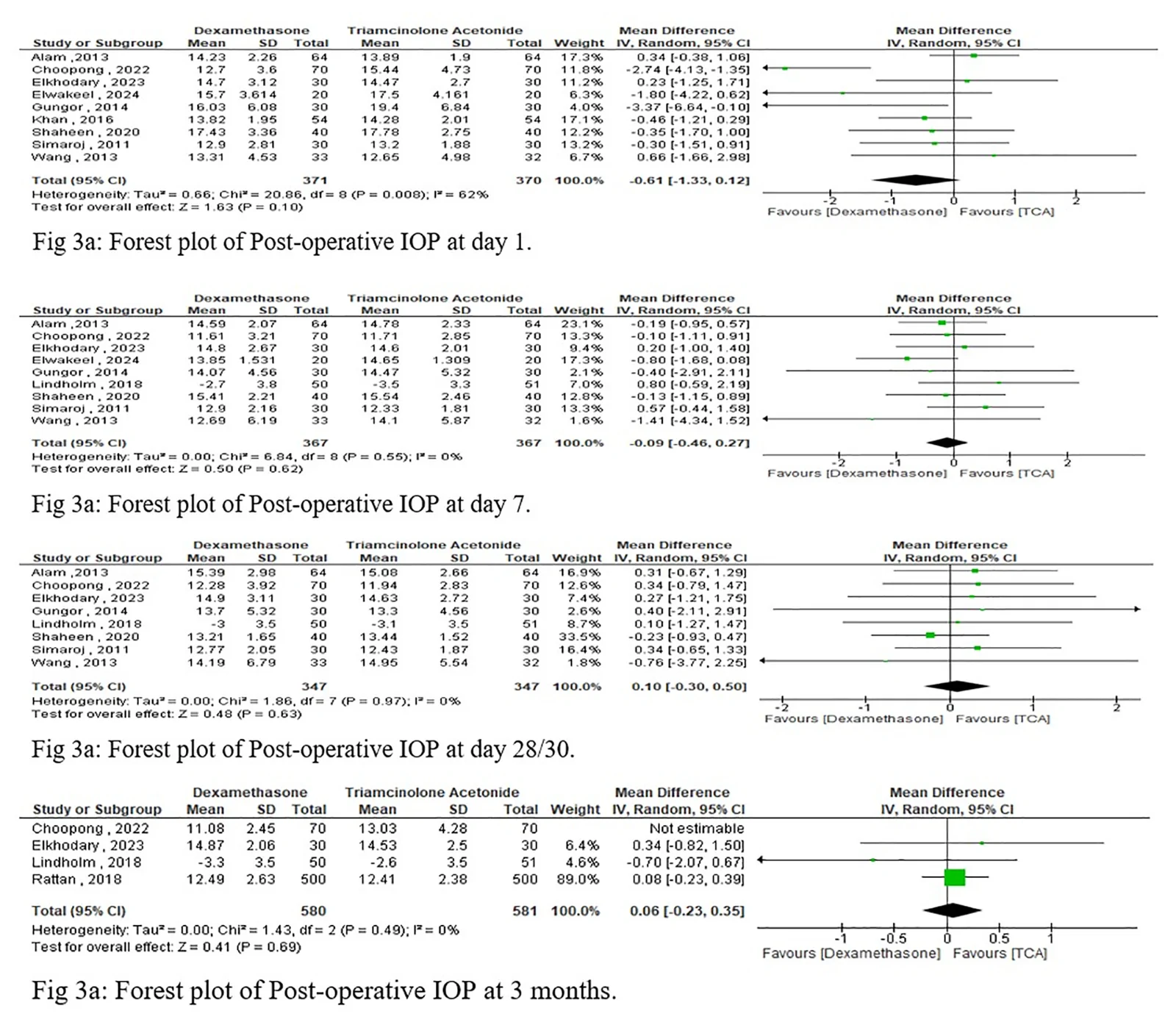
Forest plot depicting postoperative intraocular pressure at day 1, 7, 28/30 and 3 months.

- At 1 day post-op, the meta-analysis of 9 studies showed no statistically significant difference in IOP between the Dexamethasone and TCA groups (MD: −0.61 mm Hg, 95% CI: −1.33 to 0.12, *P* = .10). Sufficient heterogeneity was observed among these studies (*I*^2^ = 62%). Although not statistically significant, the MD marginally favors Dexamethasone, suggesting a slightly lower IOP in this group at the 1 day time point. Leave one out sensitivity analysis was performed for this outcome at day 1 follow-up. Removing choopong 2022 from the pooled analysis revealed a substantial decrease in heterogeneity (*I*^2^ = 20%), seen in Supplementary Figure 1, Supplemental Digital Content 1.
- At 7 days post-op, the pooled data from 9 studies indicated no significant difference between the 2 interventions (MD: −0.09 mm Hg, 95% CI: −0.46 to 0.27, *P* = .62). The studies were homogeneous for this outcome (*I*^2^ = 0%). The results marginally favor Dexamethasone, though the negligible difference is unlikely to be clinically meaningful.
- At 28/30 days post-op, the pooled results from 8 studies again demonstrated no significant difference between the groups (MD: 0.10 mm Hg, 95% CI: −0.30 to 0.50, *P* = .63). The studies were consistent, showing no heterogeneity (*I*^2^ = 0%). The analysis slightly favors TCA at this time point, but the difference remains minimal.
- At 3 months (90 days) post-op, data from 4 studies showed a nonsignificant trend, with no conclusive evidence of a difference between Dexamethasone and TCA (MD: 0.06 mm Hg, 95% CI: −0.23 to 0.35, *P* = .69). Heterogeneity was negligible for this time point (*I*^2^ = 0%). The results marginally favor TCA although the CI crosses zero, reinforcing the lack of a significant difference between the 2 treatments at 3 months.

### 3.5. Meta-analysis of secondary outcomes

#### 3.5.1. Effect of dexamethasone vs TCA on VA

The pooled analysis of LogMAR VA, where a lower score indicates better vision, at various postoperative time intervals is presented in Figure 4A–C). The results for each time point are as follows:

**Figure 4.**
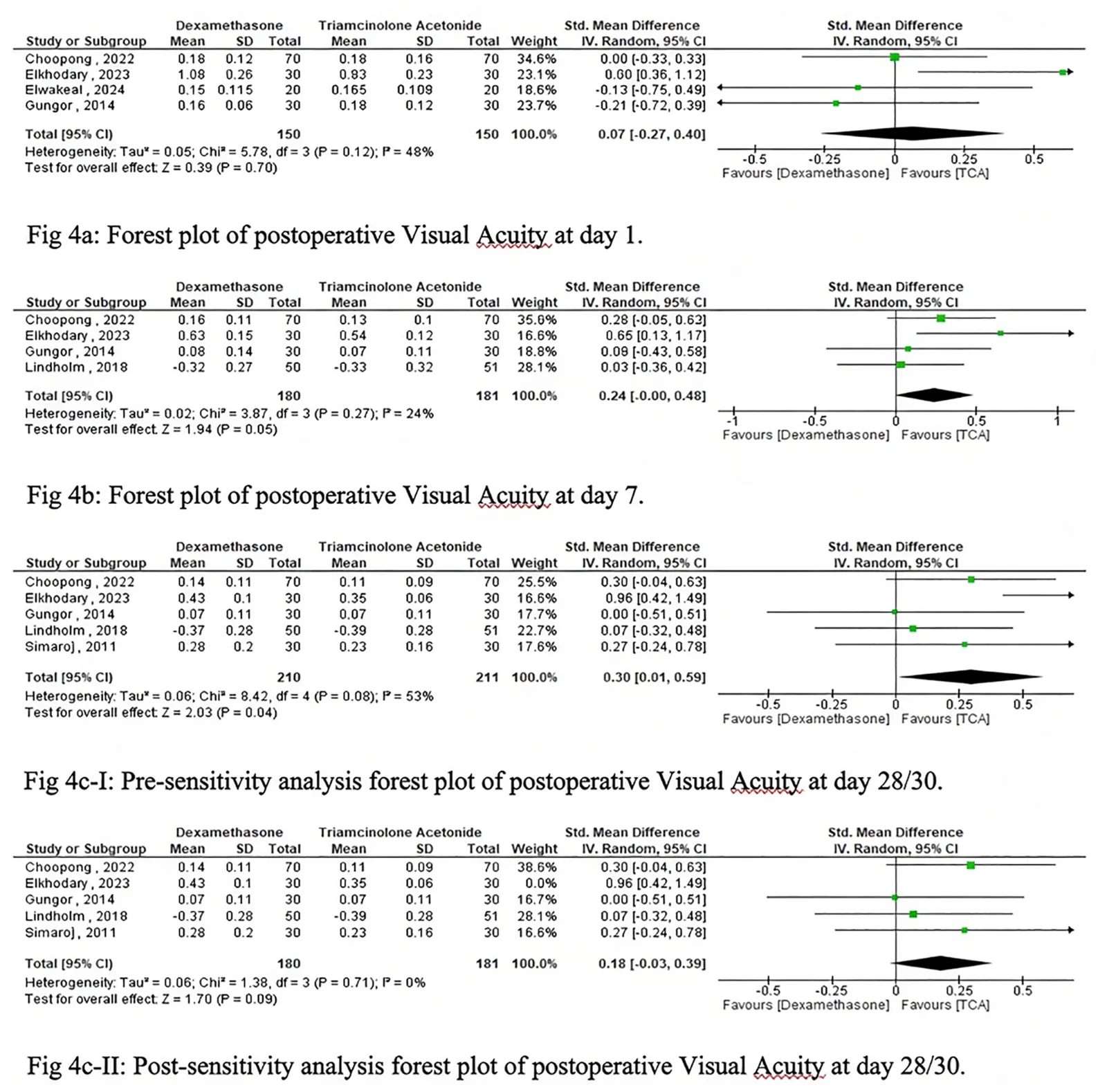
Forest plot depicting postoperative visual acquity at day 1, 7, and 28/30.

- At 1 day post-op, the meta-analysis of 4 studies showed no statistically significant difference in VA between the TCA and Dexamethasone groups (SMD: 0.07, 95% CI: −0.27 to 0.40, *P* = .70). Moderate heterogeneity was observed among these studies (*I*^2^ = 48%). The results marginally favor TCA, suggesting a slight trend toward better VS in this group at the 1 day time point.
- At 7 days post-op, the pooled data from 4 studies indicated a significant difference favoring TCA between the 2 interventions (SMD: 0.24, 95% CI: −0.00 to 0.48, *P* = .05). The heterogeneity was substantial for this outcome (*I*^2^ = 24%). The analysis favors TCA, indicating better VA in this group at 7 days postoperatively.
- At 28/30 days post-op, the analysis of 5 studies demonstrated a statistically significant difference though modest, difference favoring TCA in VA (SMD: 0.30, 95% CI: 0.01 to 0.59, *P* = .04) suggesting a meaningful improvement in VA with TCA compared to Dexamethasone at the 28/30-day time point. The studies showed considerable heterogeneity (*I*^2^ = 53%).

#### 3.5.2. Effect of dexamethasone vs TCA on anterior chamber flare

The pooled analysis of anterior chamber flare, where higher values indicate greater inflammation, across postoperative time intervals is presented in Figure 5A–C. The SMD was used to pool the data due to varying measurement scales. The results for each time point are as follows:

**Figure 5.**
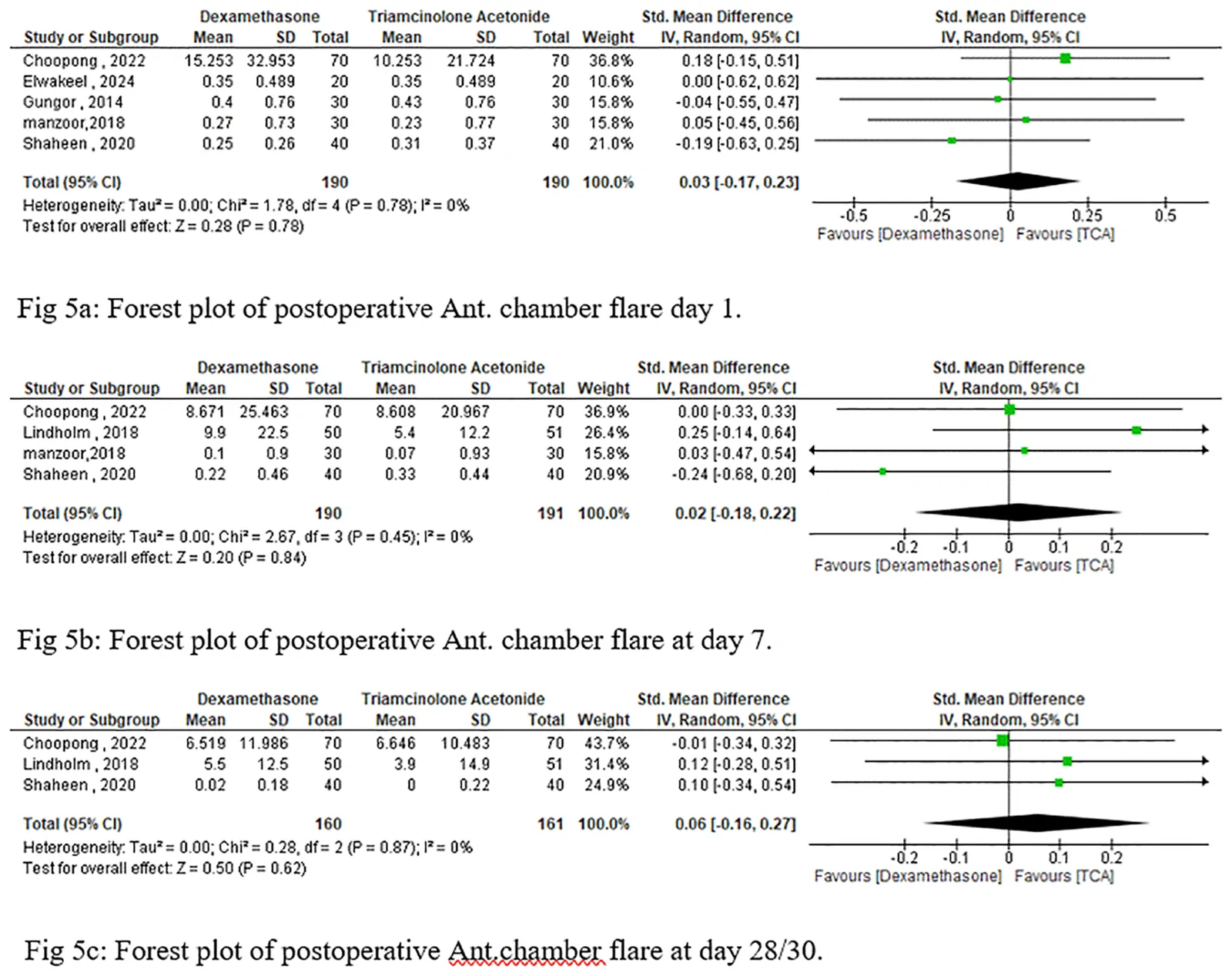
Forest plot depicting postoperative ant. Chamber flare at day 1, 7, and 28/30.

- At 1 day post-op, the meta-analysis of 5 studies showed no statistically significant difference in anterior chamber flare between the Dexamethasone and TCA groups (SMD: 0.03, 95% CI: −0.17 to 0.23, *P* = .78), with no heterogeneity (*I*^2^ = 0%). The results slightly favor TCA, suggesting a marginally lower flare, though the difference is not clinically meaningful.
- At 7 days post-op, the pooled data from 4 studies indicated a statistically nonsignificant (SMD: 0.02, 95% CI: −0.18 to 0.22, *P* = .84). The studies were homogeneous for this outcome (*I*^2^ = 0%). The analysis slightly favors TCA, showing a negligible trend toward better control of anterior chamber flare at 7 days.
- At 28/30 days post-op, the analysis of 3 studies demonstrated no statistically significant difference (SMD: 0.06, 95% CI: −0.16 to 0.27, *P* = .62), with no heterogeneity (*I*^2^ = 0%). The diamond slightly favors TCA, indicating a marginal trend toward reduced flare with this intervention, though the difference remains nonsignificant.

#### 3.5.3. Effect of dexamethasone vs TCA on anterior chamber cells

The pooled analysis of anterior chamber cells across postoperative time intervals is presented in Supplementary Figure 2, Supplemental Digital Content 2 *(a to c*). The SMD was used to pool the data. The results for each time point are as follows:

- At 1 day post-op, the meta-analysis of 5 studies showed no statistically significant difference in anterior chamber cells between the Dexamethasone and TCA groups (SMD: 0.14, 95% CI: −0.07 to 0.34, *P* = .19). The studies were homogeneous for this outcome (*I*^2^ = 0%). The results greatly favor TCA, suggesting a notable trend toward better control of inflammatory cells in the anterior chamber at the 1 day time point.
- At 7 days post-op, the pooled data from 5 studies indicated no significant difference between the 2 interventions (SMD: 0.06, 95% CI: −0.14 to 0.27, *P* = .53), with no heterogeneity (*I*^2^ = 0%). The analysis favors TCA, though the effect size is small and unlikely to be clinically significant.
- At 28/30 days post-op, the analysis of 2 studies demonstrated no statistically significant difference (SMD: −0.02, 95% CI: −0.29 to 0.24, *P* = .87), with no heterogeneity (*I*^2^ = 0%). The results slightly favor Dexamethasone at this time point, though the difference is negligible and nonsignificant.

#### 3.5.4. Effect of dexamethasone vs TCA on ECD

The pooled analysis of ECD at 7 days postoperatively is presented in Supplementary Figure 3, Supplemental Digital Content 3. The meta-analysis of 2 studies showed a nonsignificant trend towards greater endothelial cell loss in the Dexamethasone group compared to the TCA group. The MD was −0.32 cells/mm^2^ (95% CI: −0.71–0.07, *P* = .11).

This result, while not reaching statistical significance at the conventional threshold (*P* < .05), suggests a potential for greater endothelial cell damage with topical Dexamethasone compared to intracameral TCA in the early postoperative period. The studies were perfectly homogeneous for this outcome (*I*^2^ = 0%). Data for ECD at other predefined time intervals (1 day, 14 days, 28/30 days, and 3 months) were not available for pooling.

### 3.6. GRADE assessment

According to the GRADE analysis, all comparisons of Dexamethasone to TCA in cataract surgeries exhibited very low certainty of avoidance of risk. Due to this low level of certainty across each primary and secondary outcome (IOP, VA, anterior chamber flare, anterior chamber cells and ECD), the actual impact of these medications may be vastly different than what was estimated in this meta-analysis.

Regarding IOP, Dexamethasone is suggested to be no different than TCA at all postoperative time points (1 day, 7-day, 28–30 days, and 3 months); however, there is a significant degree of uncertainty surrounding this conclusion. There is also a significant degree of uncertainty concerning the use of Dexamethasone vs TCA for inflammatory markers, with Dexamethasone possibly produces higher values for anterior chamber flare and anterior chamber cells than TCA at early postoperative time points, but again the degree of certainty is very low.

The studies reviewed demonstrated that use of Dexamethasone compared to TCA may be inferior regarding visual outcomes at 7 and 28/30 days postoperatively when compared to TCA, but again, there is very low certainty associated with this conclusion. Regarding ECD, Dexamethasone may lead to greater endothelial cell loss at 7 days postoperatively, though the evidence is also very uncertain.

Imprecision (large CIs crossing zero & failing to rule out clinically beneficial or clinically harmful), risk of bias (issues with study design or execution) were the main factors leading to decreased confidence in evidence across all outcomes. In addition to these major categories of evidence limitations: potential differences between studied population, intervention, and outcome measurement were also deemed to create evidence limitations due to indirectness. These results are detailed in the Summary of Findings table (Supplementary Table 2, Supplemental Digital Content 4).

### 3.7. Publication bias

The funnel plot exhibited asymmetry, which can indicate publication bias (Supplementary Figure 4, Supplemental Digital Content 5). Egger’s regression test, which was used to formally evaluate this, did not find any statistically significant evidence of bias (*P* = .225). We conclude that there is no significant publication bias because the *P*-value is >.05, but the visual asymmetry raises the potential of unreported null research. The results should be interpreted with this limitation in mind.

## 4. Discussion

This updated systematic review and meta-analysis was conducted to compare the comparative efficacy of dexamethasone and TCA in controlling postoperative inflammation after cataract surgery. In general, both dexamethasone and TCA performed well across the included outcomes. We did not observe any consistent or statistically significant advantage of one over the other. This overall pattern is similar to what earlier studies have reported, where both agents show comparable anti-inflammatory effects.^[15]^ Despite cataract surgery being one of the most commonly performed ophthalmic procedures, postoperative inflammation still continues to remain a relevant issue. It mainly occurs due to disruption of the blood–aqueous barrier, leading to anterior chamber cells and flare.^[22]^

As discussed in the results, no meaningful differences in IOP were seen at any postoperative time point. This is in agreement with previous meta-analyses suggesting that both drugs are relatively similar in terms of pressure-related safety.^[15]^ That said, some individual studies have pointed toward a delayed change in IOP with TCA, likely because it remains in the eye for a longer duration. A slight trend in that direction was visible in our analysis at later follow-ups, but it did not reach statistical significance. Even so, it may still be reasonable to use caution in known steroid responders.^[23,24]^ This may also be due to differences in pharmacokinetics and individual steroid response, which can mask small but clinically relevant IOP changes across study populations.^[25]^

The findings for VA were somewhat mixed. There was no difference on the first postoperative day, but TCA appeared to have slightly better outcomes at 7 days and 1 month. When we performed sensitivity analysis, this difference disappeared, suggesting that it may not be a stable or reliable effect. It is possible that a few individual studies influenced this result. Most of the existing literature reports similar visual outcomes between the 2 drugs, so our findings do not substantially differ, though they hint at a possible short-term benefit with TCA that is not consistently observed.^[26]^ This variability may also reflect differences in study design, surgical techniques, and postoperative care protocols across included trials.^[15]^

When looking specifically at inflammation, including anterior chamber cells and flare, no statistically significant differences were identified at any time point. Interestingly, the direction of effect often leaned slightly toward TCA, even early after surgery. This is somewhat unexpected, as dexamethasone is generally thought to act more rapidly. One possible explanation could be the sustained intraocular presence of TCA, which might provide a steadier anti-inflammatory effect from the beginning. Still, since these differences were not significant, they should be interpreted cautiously.^[27]^ Another contributing factor could be heterogeneity in inflammation assessment and postoperative regimens across studies, which may reduce comparability of outcomes.^[28]^

Our analysis showed a trend toward greater endothelial cell loss in the dexamethasone group; however, this difference was not statistically significant. In contrast, the study by Sali et al reported worse outcomes in the TCA group. This inconsistency may be explained by differences in study design, patient populations, dosing regimens, and duration of follow-up, all of which can influence endothelial cell measurements. Given the lack of statistical significance in our findings and the variability across studies, it remains difficult to draw firm conclusions regarding the comparative effects of these agents on corneal endothelial health. Further well-designed studies are needed to better clarify this relationship.^[29]^

In clinical practice, the decision of opting between dexamethasone and TCA often depends more on the patient’s compliance than on any major difference in drug’s mechanism or efficacy. TCA can be useful in patients who have problems with adhering to repetitive postoperative eye drops, as it is long lasting and allows for sustained control with a single administration. In contrast, dexamethasone may be a better choice when more precise control over dosing is needed, or in situations where treatment might need to be adjusted or stopped quickly because of side effects.^[30]^ This solidifies the hypothesis that both dexamethasone and TCA continue to be reliable options for controlling postoperative inflammation after cataract surgery, which aligns with findings reported in the wider literature.^[31]^

### 4.1. Strengths and limitations

This meta-analysis has several notable advantages. It consists of 12 RCTs, ensuring a high level of evidence while minimizing the potential for selection bias. The large total sample size enhances the statistical power and reliability of the pooled results. Additionally, the included studies demonstrated high consistency, with minimal heterogeneity (*I*^2^ < 50%) across significant outcomes like IOP, anterior chamber inflammation, and ECD. The inclusion of sensitivity analyses further strengthened the validity of the findings. However, the available evidence is limited by variations in the administration route and dosage across studies, which may have influenced the results. Moreover, most studies had relatively short follow-up durations, often <3 months, which restricted the ability to evaluate long-term safety and sustained efficacy. The limited sample sizes in some trials may also reduce the statistical power to detect minor but clinically relevant differences. Although Egger’s test did not reach statistical significance (*P* = .225), the visible asymmetry in the funnel plot suggests that publication bias cannot be completely excluded. Furthermore, 2 studies had a high risk of bias (Wang et al^[32]^ Simaroj et al^[33]^) primarily due to deviations from intended interventions and outcome measurements. Additionally, several pooled estimates combined RCTs and non-randomized prospective studies. While this increases sample size, it may reduce certainty of evidence. We therefore downgraded GRADE ratings for these outcomes and caution that conclusions should be interpreted in light of mixed study designs. Lastly, while this meta-analysis included 5 newly published RCTs, one study by Elkhodary et al^[34]^ was a non-RCT, which may introduce selection bias and reduce the overall methodological uniformity of the pooled evidence. To ensure the long-term safety of the corticosteroids, future research should focus on larger, multicenter RCTs that employ standardized outcome reporting, extend follow-up periods and last but not the least further investigations are needed to assess patient-reported outcomes, such as adherence and comfort, as well as to conduct pharmacoeconomic comparisons between topical and intracameral corticosteroid dosages in standard cataract surgery practices.

## 5. Conclusion

In conclusion, both dexamethasone and TCA are effective and generally safe for controlling inflammation after cataract surgery. Dexamethasone provides a faster onset of action, whereas TCA offers a longer duration and may reduce dependence on patient compliance. However, these findings should be interpreted with caution because several pooled analyses combined randomized and non-randomized studies, and the certainty of evidence remains low. The corrected use of MD (mm Hg) for IOP outcomes ensures methodological consistency, but residual heterogeneity across study designs may influence the pooled estimates. Clinical decisions should therefore be individualized, guided by patient needs and clinician expertise. This updated meta-analysis provides a more current evidence base while acknowledging the limitations inherent in the available data.

## Author contributions

**Conceptualization:** Humayou Ahmed.

**Data curation:** Humayou Ahmed.

**Formal analysis:** Humayou Ahmed.

**Funding acquisition:** Muhammad Saad Khan, Wajeeha.

**Investigation:** Muhammad Saad Khan, Samreen Shahid, Zainab Arif, Wajeeha, Maheen Hamid.

**Methodology:** Muhammad Saad Khan, Zauha Fawad Memon, Samreen Shahid, Zainab Arif, Wajeeha, Maheen Hamid.

**Project administration:** Zauha Fawad Memon, Samreen Shahid, Zainab Arif, Muhammad Mohsin Khan, Daniyal Zulfiqar.

**Software:** Zauha Fawad Memon, Serish Bano, Syed Ali Hassan, Shifa, Muhammad Mohsin Khan.

**Supervision:** Serish Bano, Syed Ali Hassan, Shifa, Manal Al-Dhaheri, Humayou Ahmed.

**Validation:** Serish Bano, Syed Ali Hassan, Shifa, Muhammad Mohsin Khan, Manal Al-Dhaheri, Humayou Ahmed.

**Visualization:** Syed Ali Hassan, Umarah Lutuf, Aqsa, Manal Al-Dhaheri.

**Resources:** Shifa, Daniyal Zulfiqar.

**Writing – original draft:** Umarah Lutuf, Aqsa, Daniyal Zulfiqar, Maheen Hamid, Manal Al-Dhaheri.

**Writing – review & editing:** Umarah Lutuf, Aqsa, Daniyal Zulfiqar, Maheen Hamid, Manal Al-Dhaheri.












