# Supplementary material for: Dexamethasone versus triamcinolone acetonide for the management of postoperative inflammation following cataract surgery: A systematic review and meta-analysis
Source: Medicine (Baltimore). 2026 Oct 9;105(41):e50840. doi: 10.1097/MD.0000000000050840 (PMC13657535; doi:10.1097/MD.0000000000050840)
Supplement: Supplementary file 1 [file medi-105-e50840-s004.docx]

| **Summary of findings:** | | | | | | |
| --- | --- | --- | --- | --- | --- | --- |
| **Dexamethasone compared to TCA following Cataract Surgery** | | | | | | |
| **Patient or population:** Cataract surgery  **Intervention:** Dexamethasone  **Comparison:** TCA | | | | | | |
| Outcomes | **Anticipated absolute effects^*^** (95% CI) | | Relative effect (95% CI) | № of participants (studies) | Certainty of the evidence (GRADE) | Comments |
|  | **Risk with TCA** | **Risk with Dexamethasone** |  |  |  |  |
| Post-operative IOP at day 1 | - | MD **0.61 lower** (1.33 lower to 0.12 higher) | MD -0.61 (-1.33,0.12) | 741 (8 RCTs+ 1NRCT) | ⨁◯◯◯ Very low^a,b,c,d^ | Dexamethasone may reduce/have little to no effect on post-operative IOP at day 1 but the evidence is very uncertain. |
| Post-operative IOP at day 7 | - | MD **0.09 lower** (0.46 lower to 0.27 higher) | MD -0.09 (-0.46,0.27) | 734 (7 RCTs+2 NRCTs) | ⨁◯◯◯ Very low^e,f,g,h,i^ | Dexamethasone may reduce/have little to no effect on post-operative IOP at day 7 but the evidence is very uncertain. |
| Post-operative IOP at day 28/30 | - | MD **0.1 higher** (0.3 lower to 0.5 higher) | MD 0.1 -(0.3,0.5) | 694 (6 RCTs+2 NRCTs) | ⨁◯◯◯ Very low^j,k,l^ | Dexamethasone may increase/have little to no effect on post-operative IOP at day 28/30 but the evidence is very uncertain. |
| Post-operative IOP at 3 months | - | MD **0.06 higher** (0.23 lower to 0.35 higher) | MD 0.06 (-0.23,0.35) | 1161 (3 NRCTs) | ⨁◯◯◯ Very low^m,n,o^ | Dexamethasone may increase/have little to no effect on post-operative IOP at 3 months but the evidence is very uncertain. |
| Post-operative Visual Acuity (VA) at day 1 | - | SMD **0.07 higher** (0.27 lower to 0.4 higher) | SMD 0.07 (-0.27,0.40) | 300 (3 RCTs+1 NRCT) | ⨁◯◯◯ Very low^p,q,r,s^ | Dexamethasone may increase/have little to no effect on post-operative Visual Acuity (VA) at day 1 but the evidence is very uncertain. |
| Post-operative Visual Acuity (VA) at day 7 | - | SMD **0.24 higher** (0 to 0.48 higher) | SMD 0.24 (-0.00,0.48) | 361 (2 RCTs+2 NRCTs) | ⨁◯◯◯ Very low^t,u,v^ | Dexamethasone may increase/have little to no effect on post-operative Visual Acuity (VA) at day 7 but the evidence is very uncertain. |
| Post-operative Visual Acuity (VA) at day 28/30 | - | SMD **0.3 higher** (0.01 higher to 0.59 higher) | SMD 0.30 (0.01,0.59) | 421 (2 RCTs+2 NRCTs) | ⨁◯◯◯ Very low^w,x,y,z^ | Dexamethasone may increase/have little to no effect on post-operative Visual Acuity (VA) at day 28/30 but the evidence is very uncertain. |
| Post-operative Ant.chamber flare at day 1 | - | SMD **0.03 higher** (0.17 lower to 0.23 higher) | SMD 0.03 (-0.17,0.23) | 380 (5 RCTs) | ⨁◯◯◯ Very low^aa,ab,ac^ | Dexamethasone may increase/have little to no effect on post-operative Ant.chamber flare at day 1 but the evidence is very uncertain. |
| Post-operative Ant.chamber flare at day 7 | - | SMD **0.02 higher** (0.18 lower to 0.22 higher) | SMD 0.02 (-0.18,0.22) | 381 (3 RCTs+1 NRCT) | ⨁◯◯◯ Very low^ad,ae,af^ | Dexamethasone may increase/have little to no effect on post-operative Ant.chamber flare at day 7 but the evidence is very uncertain. |
| Post-operative Ant.chamber flare at 28/30 day | - | SMD **0.06 higher** (0.16 lower to 0.27 higher) | SMD 0.06 (-0.16,0.27) | 321 (2 RCTs+NRCT) | ⨁◯◯◯ Very low^ag,ah,ai^ | Dexamethasone may increase/have little to no effect on post-operative Ant.chamber flare at 28/30 day but the evidence is very uncertain. |
| Post-oeprative Ant.chamber cells at day 1 | - | SMD **0.14 higher** (0.07 lower to 0.34 higher) | SMD 0.14 (-0.07,0.34) | 380 (5 RCTs) | ⨁◯◯◯ Very low^aj,ak,al^ | Dexamethasone may increase/have little to no effect on post-oeprative Ant.chamber cells at day 1 but the evidence is very uncertain. |
| Post-operative Ant.chamber cells at day 7 | - | SMD **0.06 higher** (0.14 lower to 0.27 higher) | SMD 0.06 (-0.14,0.27) | 380 (5 RCTs) | ⨁◯◯◯ Very low^am,an,ao^ | Dexamethasone may increase/have little to no effect on post-operative Ant.chamber cells at day 7 but the evidence is very uncertain. |
| Post-operative Ant.chamber cells at day 28/30 | - | SMD **0.02 lower** (0.29 lower to 0.24 higher) | SMD -0.02 (-0.29,0.24) | 220 (2 RCTs) | ⨁◯◯◯ Very low^ap,aq,ar^ | Dexamethasone may reduce/have little to no effect on post-operative Ant.chamber cells at day 28/30 but the evidence is very uncertain. |
| Post-operative Endothelial cell density (ECD) at day 7 | - | SMD **0.32 lower** (0.71 lower to 0.07 higher) | SMD -0.32 (-0.71,0.07) | 105 (2 RCTs) | ⨁◯◯◯ Very low^as,at,au^ | Dexamethasone may reduce/have little to no effect on post-operative Endothelial cell density (ECD) at day 7 but the evidence is very uncertain. |

**Supplementary table 2**: Grade Assessment table
