# Supplementary material for: Dexamethasone versus triamcinolone acetonide for the management of postoperative inflammation following cataract surgery: A systematic review and meta-analysis
Source: Medicine (Baltimore). 2026 Oct 9;105(41):e50840. doi: 10.1097/MD.0000000000050840 (PMC13657535; doi:10.1097/MD.0000000000050840)
Supplement: Supplementary file 2 [file medi-105-e50840-s006.docx]

 **Supplementary table 1: Newcastle-Ottawa Scale**
